# Fructan and hormone connections

**DOI:** 10.3389/fpls.2015.00180

**Published:** 2015-03-23

**Authors:** Ravi Valluru

**Affiliations:** Global Wheat Program, International Maize and Wheat Improvement Center (CIMMYT)El Batan, Mexico

**Keywords:** abscisic acid, auxins, ethylene, fructans, hormones, reserve carbohydrates, sugars

Plants rely on “reserve” (stored) carbon (C) for growth and survival when newly synthesized C becomes limited. Besides a classic yet recalcitrant C reserve starch, fructans, a class of sucrose-derived soluble fructosyl-oligosaccharides, represent a major store of C in many temperate plant species including the economically important Asteraceae and Poaceae families (Hendry, 1993). Dicots typically accumulate inulin-type fructans as long-term storage (underground organs) whilst grasses and cereals accumulate fructans as short-term reserves in above-ground parts (Pollock and Cairns, 1991; Van Laere and Van den Ende, 2002). Unlike chloroplast-based water-insoluble starch, fructans are semi-soluble, possess flexible structures (Phelps, 1965; Valluru and Van den Ende, 2008), can be synthesized at low temperatures (Pollock and Cairns, 1991), and are degraded by a single type of fructan hydrolases, fructan exohydrolases (FEHs). Unlike starch that store in plastids, fructans store in vacuoles, which is physically less stressful to the active constituents of, and allows more C synthesis by, the photosynthetic cell, which may be different in dicots where fructans do not typically accumulate in green parts.

Plants synthesize diverse fructan types exhibiting a wide range of functions (for review, see Valluru and Van den Ende, 2008; Van den Ende, 2013). Fructan biosynthetic enzymes, fructosyltransferases (FTs), which evolved from vacuolar-type acid invertases (VIs) (Altenbach et al., 2009), use sucrose (Suc) as a substrate whereby an organ-specific Suc threshold triggers FT genes at the transcriptional level (Lu et al., 2002). Though the regulatory mechanism of Suc signal transduction remains largely elusive, transcription factors (TFs) can be suspected to mediate such inductive processes either by directly binding and stimulating FT genes (e.g., TaMYB13 TF binds to FT genes, 1-SST and 6-SFT; Xue et al., 2011) or by up-regulating vacuolar based proteins (e.g., TaMYB13 TF up-regulates vacuolar processing enzyme, Taγ-VPE1, whose mRNA levels highly correlated with FTs mRNA levels in wheat stems; Kooiker et al., 2013).

In addition, protein phosphatases (PP2A; Martínez-Noël et al., 2009) and second messenger Ca^2+^ (Martínez-Noël et al., 2006) mediate Suc-induction of fructan synthesis in wheat, although the underlying mechanisms remain largely undefined. The cationic role of Ca^2+^ in fructan synthesis is somewhat counterintuitive because Suc induces a Ca^2+^ efflux from the vacuole (Furuichi et al., 2001), the site of fructan synthesis. Perhaps Suc might ensure more alkaline (less acidic) vacuolar environment [Suc-induces Slowly activating Vacuolar (SV) ion channel that transiently effluxes vacuolar Ca^2+^; (Pottosin and Schönknecht, 2007)], favoring fructan synthesis that is thought to be less stable under low pH (Flores-Maltos et al., 2014). Some of the protein mediators involved in Suc-mediated induction of fructan synthesis, including Ca^2+^ signaling components, calmodulin (CaM), calcineurin B-like (CBL1), and Ca^2+^–dependent protein kinases (CDPKs), are closely involved in hormone signaling and environmental stress (Ludwig et al., 2004).

Remarkable progress has been made in understanding the crosstalk between sugar signaling and hormonal networks (León and Sheen, 2003; Matsoukas, 2014). Abscisic acid (ABA) appears to have positive effect on reserve C storage, whereby ABA greatly enhances Suc-induction of starch biosynthetic genes, ADP-glucose pyrophosphorylase (subunit ApL3) and starch-branching enzyme Sbe2.2, with no effect on their expression in the absence of Suc (Rook et al., 2001), implying that ABA promotes reserve starch biosynthesis. Similarly, ABA (50 mM) has recently been shown to promote fructan accumulation by increasing gene expressions of 1-FFT and 1-SST in agave (Suárez-González et al., 2014). Further, the barley FT gene (6-SFT) promoter carries recognition sites for MYC, MYB proteins and many *cis*-acting elements that mediate ABA and drought responses (Nagaraj, 2004). Indeed, hormonal regulation of fructan metabolic enzymes (1-SST and 1-FEHI) has already been suggested by Bausewein et al. (2012). ABA actions are likely to be further promoted by a small Ca^2+^ sensor, CBL1, which represses PP2C, a negative component of ABA signaling (Lan et al., 2011).

It is also likely that the low concentrations of ethylene (ET) and auxins (AUX) contribute to promote fructan storage. Supportively, low concentrations of exogenous 1-aminocyclopropance 1-carboxylicacid (ACC at 1 μM, ethylene precursor) and AUX (13.4 μM) increased fructan content in 2-month-old agave (Barreto et al., 2010). Further, a high concentration (10 μM or 10 μL L^−1^) of exogenous ET decreased fructan content in onion, which was counteracted by ethylene binding inhibitor 1-methylcyclopropene (1-MCP) (Cools et al., 2011). Indeed, the hormonal balance (AUX/cytokinins, CKs) has been suggested to play a role in the regulation of fructan synthesizing enzymes (1-SST and 1-FFT) in *Vernonia herbacea* (Trevisan et al., 2014). Presumably, FTs could carry motifs for these hormones, as reported for FEHs that carry motifs for AUX, ABA, ET, gibberellins (GA), and salicylic acid (SA) (Michiels et al., 2004). Both AUX and ET homeostasis could be regulated by Ca^2+^ signaling. Ca^2+^/CaM binds to small AUX-up RNAs (SAURs) proteins (Yang and Poovaiah, 2000), the negative regulators of AUX synthesis (Kant et al., 2009). In contrast, AUX induces SAURs as a feed-forward mechanism (Kant et al., 2009). In addition to CDPKs (that induces ACC synthases, ACS2 and ACS6), PP2As tightly control ET biosynthesis by differentially regulating the turnover of ACS5 and ACS6 isoforms (Skottke et al., 2011). Taken all together, it appears that hormones may act at low concentration either as an “inductive signal” or as a “facilitator” mediating Suc-induction of fructan biosynthesis, a presumption worthy of further attention (Figure 1A). Recent studies emphasize a close relation between the spatio-temporal dynamics of hormones (ET and ABA) and fructan synthesis and its related gene transcripts in the endosperm transfer cells of barley (Thiel et al., 2012; Peukert et al., 2014).

**Figure 1:**
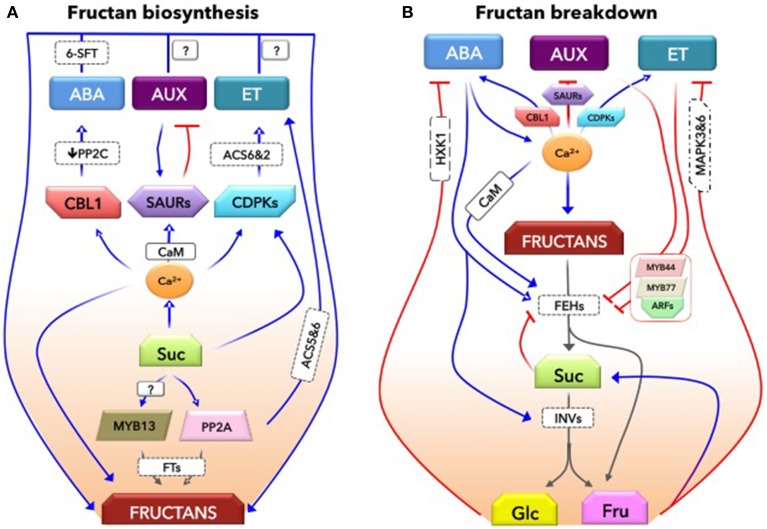
**A conceptual mechanistic basis for the interaction between fructans and hormones**. This model explains how hormones may closely regulate both biosynthesis **(A)** and catabolism **(B)** of fructans. **(A)**, The involvement of hormones in fructan biosynthesis. Suc-specific pathways involving transcription factors (MYB13), protein phosphatase (PP2A), and calcium (Ca^2+^) signaling closely mediate Suc-induction of fructan biosynthesis. In addition, these components are involved in hormones, abscisic acid (ABA), auxins (AUX), and ethylene (ET) biosynthesis as well as their homeostasis. A calcium sensor, CBL1, promotes ABA signaling by repressing the negative regulator of ABA signaling, PP2C. Ca^2+^/CaM module activates small AUX-up RNA proteins (SAURs) that control AUX levels. In contrast, AUX positively regulates SAURs. While Ca^2+^–dependent protein kinases (CDPKs) control the ET biosynthesis; PP2A tightly control ET biosynthesis by differentially regulating the turnover of ACC synthase (ACS5 and 6) isoforms. The presence of relatively stable, low levels of these hormones is important, and may form part of Suc-specific pathways positively regulating fructan biosynthesis via fine-tuning of FTs. **(B)**, The hormonal network regulating fructan degradation. Stress-inducible ABA induces fructan catabolic enzymes, fructan exohydrolases (1-FEH), promoting fructan degradation in wheat. Suc is further hydrolysed by ABA-induced acid invertases (INVs). Part of the Fructose (Fru) would be used for Suc synthesis. While glucose (Glc) antagonizes ABA signaling, Fru antagonizes ET signaling, mediated by mitogen-activated protein kinases (MAPK3 and 6). It is known that ET counteracts ABA functions. In order to promote ET signaling, it is suggested here that both ET and AUX signaling that function synergistically may counteract ABA signaling and repress 1-FEH expression, thereby reducing glucose and fructose levels. The timing of fructan degradation may be a critical process affecting carbon availability and on-going physiological processes that could indeed alter the initiation of leaf senescence. Such AUX/ET signaling repression of 1-FEH might be mediated by a transcriptional complex MYB44-MYB77. This protein complex interacts with auxin response factors (ARFs) that bind to auxin responsive elements (AuxRE) of auxin responsive genes as was shown in arabidopsis. While ABA enhances Ca^2+^ levels, which in turn promotes ABA signaling and enhances FEH activity, Ca^2+^ signaling-based CDPKs (via ACS6 and 2) also promote ET biosynthesis. This suggests that a subtle hormonal balance governs biological processes more sensibly than could achieve by a single hormone. However, this conceptual model is largely constructed based on the knowledge from Arabidopsis; hence, this needs to be confirmed in fructan-accumulating species. Blue lines represent positive regulation while red lines indicate negative regulation. Gray lines depict degradation processes. See text for further explanations and the references.

This, however, raises a subsequent question: “Do hormones also regulate fructan catabolism?” (Figure 1B), considering that (1) hormones coordinately regulate stress-inducible acid INVs (e.g., *Ivr2*; Trouverie et al., 2004) and hexose transporters (VvHT5; Hayes et al., 2010) regulating carbon partitioning and sink strength; (2) FEHs evolved from acid INVs (INVs) of cell-wall type (Le Roy et al., 2007) and Suc directly inhibits FEH activities at the enzyme level (Verhaest et al., 2007); and (3) FEHs (FEHIIa) gene promoter carry motifs for several hormones such as AUX (ASF1, ARF, and CATATGGMSAUR), ABA (ABRE), ET (GC box and ERE), GA (GARE), and SA (ASF1 and W box) (Michiels et al., 2004). While hormones (ABA, GA, AUX, CKs, and brassinosteroids; for review, see Roitsch et al., 2003) induce extracellular INVs, one remaining question would be: “What actually triggers the induction of vacuolar FEHs under stress?.”

While stress stimuli induce FEHs (Michiels et al., 2004; Yang et al., 2004) as well as the involvement of sugar signaling after defoliation (Lothier et al., 2010), one important candidate that fulfills this role could be “stress-inducible ABA.” Recent studies are now elucidating the multiple molecular connections in how the spatial and temporal regulation of hormones permits the fine-tuning of the stress response (Dubois et al., 2013). Presumably, ABA (stress-induced or a stress-like condition) may regulate FEHs in two-ways: directly, ABA can regulate 1-FEH at the transcriptional level (Ruuska et al., 2007); or indirectly, via ABA-induced luminal acidification [ABA induces vacuolar H^+^-ATPase activity in ice plant (*Mesembryanthemum crystallinum*, Barkla et al., 1999); or increases phosphatidylinositol 3,5-bisphosphate [PtdIns(3,5)P_2_] that activates vacuolar H^+^-PPase activity in fava bean, (Bak et al., 2013)], which may act as a trigger, providing an acidic environment enhancing FEH activity as FEHs have an acidic pH optimum (~5.0) (Henson, 1989; Henson and Livingston, 1996; Van den Ende et al., 2003). ABA induction of 1-FEH may be mediated by protein kinases (e.g., PI3K signaling has been proposed to mediate ABA induction of 1-FEH1, Bausewein et al., 2012). This also suggests a possible role for pH signaling in FEH regulation. In addition, GA might be important hormone for FEH regulation (Morvan et al., 1997). Such hormone regulation of FEHs also supports the current understanding that hormones may function as “mediators” enabling the communication and transduction of environmental changes into stress responses (Pozo et al., 2015).

While fructan-based Glc (growth signaling) antagonizes ABA signaling (Morita-Yamamuro et al., 2004), fructose (Fru) signaling negatively interacts with ET signaling via mitogen-activated protein kinases (MAPK3 and 6) (Shahri et al., 2014). It therefore appears that ABA has a role in both FT and FEH induction, raising a question: “How does ABA induce both FTs and FEHs?” One suggestion is that high and low ABA levels could have opposing functions, with low concentrations promoting FTs activity whilst high concentrations induce FEHs. Such a dual role for ABA has been previously recognized in the regulation of root growth, whereby low concentration (1 μM) of ABA stimulated while high concentration (100 μM) inhibited, the root elongation growth of pea (Gaither et al., 1975).

Fructan-hormone interactions can be further envisaged by the fact that both fructans and inactive ABA form (ABA and hydroxyl ABA are conjugated with Glc for inactivation and the predominant conjugated form is ABA glucosyl ester, ABA-GE) are stored in vacuoles (Dietz et al., 2000). Apart from *de novo* biosynthesis of ABA in the cytoplasm/plastids, its conjugation/deconjugation critically controls cellular ABA levels. ABA-GE is stored in vacuoles and the apoplast, and is hydrolysed by β-glucosidases (BGs) (Dietz et al., 2000). Of the two BGs isolated so far in plants, BG2 was found in the vacuole (Xu et al., 2012). Abiotic stress normally induces BG2 activity (Xu et al., 2012) as well as tonoplast-vesicle-derived exocytosis (TVE, Valluru et al., 2008), exporting both fructans and ABA into the apoplast for further systemic signaling. A direct signaling capacity for fructans in immune modulation, mediated by Toll-Like Receptor (TLR) signaling, has recently been shown in animal cells (Vogt et al., 2013). It would be interesting to investigate such fructan signaling roles as well as fructan putative sensors (putative proteins with similar functions) in fructan-accumulating plants.

While hormones are known to be involved in C partitioning, their interaction mechanisms are largely undetermined. Since hormones extensively interact with each other and regulate biological processes accordingly, one would expect that ABA induction of 1-FEH might be subject to the actions of other hormones. As both AUX and ET signaling generally counteract ABA signaling at the whole plant level depending on their concentrations (Rock and Sun, 2005; Arc et al., 2013), these hormones can be expected to counteract ABA-induced 1-FEH expression (Zhang et al., 2014). Multiple hormones may therefore coordinately regulate 1-FEHs, whereby 1-FEH gene promoters (e.g., *1-FEH w3* in wheat) carrying motifs for AUX signaling (AUX response elements, AuxRE) show less sensitivity to ABA. Conversely, 1-FEHs that do not carry or modify motifs for AUX signaling show enhanced sensitivity to ABA, initiating early fructan degradation (Zhang et al., 2014). A similar interaction between ABA and ET signaling can be envisaged in 1-FEH regulation (Figure 1B).

The underlying mechanism of how AUX/ET networks counteract ABA function in 1-FEH regulation is unknown. It can however be suspected that several TFs might mediate such a process. Auxin response factors (ARFs), which bind to AuxRE, are classified as either transcriptional activators or repressors of AUX-responsive genes (Guilfoyle and Hagen, 2007). In epidermal and root cells of onion, a fructan-accumulating plant, ARF7 has been shown to interact with MYB77 TF, modulating AUX responsive genes (Shin et al., 2007). The process how such an interaction (MYB77 with ARF7) leads to 1-FEH repression rather than its promotion has yet to be elucidated.

It is suspected here that such a “repressive” function may have evolved due to MYB77 interaction with other R2R3 type TF, MYB44 that share partially redundant functions, and form a heterodimer complex in Arabidopsis (Jaradat et al., 2013). MYB44 carries a putative transcriptional repression motif “***E****thylene responsive element binding factor-associated* ***A****mphiphilic* ***R****epression* (EAR)” identified in members of the ethylene-responsive element binding factor, C_2_H_2_, and AUX/indole-3-acetic acid families of transcriptional regulators (Kagale et al., 2010). Of the 219 “EAR” motif proteins identified in Arabidopsis, approximately 40% of these act as negative regulators of gene expression (Kagale et al., 2010). Thus, it can be suspected that the protein complex MYB44–MYB77, may act as a negative regulator of 1-FEH, inhibiting fructan degradation in fructan-accumulating plants. In contrast, 1-FEH lacking AuxRE do not bind with this protein complex and show enhanced sensitivity to ABA, resulting in early fructan degradation. Such a dualistic regulation of hormone interaction on 1-FEH was recently demonstrated by Zhang et al. (2014).

Interestingly, ARF7 and ARF19, which are induced by ET, play critical roles in ET responses in Arabidopsis roots (Li et al., 2006), suggesting that, perhaps, ET signaling also utilizes a similar pathway, and function in concert with AUX, to counteract ABA induced 1-FEH expression. Recently, CKs have been proposed to antagonize ABA induction of 1-FEHI in chicory hairy root cell culture (Bausewein et al., 2012). However, the above conceptual model is largely constructed based on the knowledge from Arabidopsis; hence, this model needs to be tested and confirmed in fructan-accumulating species.

The study of Zhang et al. (2014) further extends excessive crosstalk between hormones and sugars to fructans, which could prove critical, especially in temperate food cereals such as wheat. Fructan-based C partitioning to developing grain has conventionally been seen as a major function of fructans especially under stress. In this regard, early fructan degradation could support on-going growth and grain-fill processes, potentially delaying leaf senescence that could extend leaf photosynthetic capacity. It is widely accepted that delayed, but not unfavorably-induced, leaf senescence enhances crop yields. Hence, the molecular marker of *1-FEH w3* could be useful for the genetic selection for high stem-reserve C partitioning and high grain-weight in wheat breeding. This study, therefore, not only rules out the imprudent notion, “*hormones are too complicated to be used for crop improvement*” but also paves the way for “*elegant utilization of hormones in improving future food security*.” Understanding such hormonal signatures in “reserve C partitioning,” one of the key traits of the conceptual model of yield (Reynolds et al., 2009), are crucial to simultaneously improve crop grain-fill and lodging resistance.

## Conflict of interest statement

The author declares that the research was conducted in the absence of any commercial or financial relationships that could be construed as a potential conflict of interest.

## Abbreviations

1-MCP: 1-methylcyclopropene
1-FFT: fructan:fructan 1-fructosyltransferase
1-SST: sucrose:sucrose 1-fructosyltransferase
6-SFT: sucrose:fructan 6-fructosyltransferase
1-FEH: 1-fructan exohydrolase
ABA-GE: ABA glucosyl ester
ABA: abscisic acid
ABRE: ABA-responsive element
ACC: 1-aminocyclopropane 1-carboxylicacid
ARFs: auxin response factors
AUX: auxins
AuxRE: auxin response element
AcINVs: acid invertases
BG: β-glucosidases
CaM: calmodulin
CBL1: calcineurin B-like
CDPK: calcium-dependent protein kinase
ET: ethylene
FEHs: fructan exohydrolases
FTs: fructosyltransferases
Fru: fructose
Glc: glucose
PP2A: protein phosphatase 2A
PP2C: protein phosphatase 2C
Suc: sucrose
TF: transcription factor
VIs: vacuolar invertases.
